# Bolide impact triggered the Late Triassic extinction event in equatorial Panthalassa

**DOI:** 10.1038/srep29609

**Published:** 2016-07-08

**Authors:** Tetsuji Onoue, Honami Sato, Daisuke Yamashita, Minoru Ikehara, Kazutaka Yasukawa, Koichiro Fujinaga, Yasuhiro Kato, Atsushi Matsuoka

**Affiliations:** 1 Department of Earth and Environmental Sciences, Kumamoto University, 2-39-1 Kurokami, Kumamoto 860-8555, Japan; 2 Japan Agency for Marine-Earth Science and Technology (JAMSTEC), 2-15 Natsushima-cho, Yokosuka 237-0061, Japan; 3 Centre for Advanced Marine Core Research, Kochi University, B200 Monobe, Nankoku 783-8502, Japan; 4 Department of Systems Innovation, School of Engineering, The University of Tokyo, 7-3-1 Hongo, Bunkyo-ku, Tokyo 113-8656, Japan; 5 Frontier Research Centre for Energy and Resources (FRCER), School of Engineering, The University of Tokyo, 7-3-1 Hongo, Bunkyo-ku, Tokyo 113-8656, Japan; 6 Department of Geology, Niigata University, Igarashi 2-no-cho 8050, Niigata 950-2181, Japan

## Abstract

Extinctions within major pelagic groups (e.g., radiolarians and conodonts) occurred in a stepwise fashion during the last 15 Myr of the Triassic. Although a marked decline in the diversity of pelagic faunas began at the end of the middle Norian, the cause of the middle Norian extinction is uncertain. Here we show a possible link between the end-middle Norian radiolarian extinction and a bolide impact. Two palaeoenvironmental events occurred during the initial phase of the radiolarian extinction interval: (1) a post-impact shutdown of primary and biogenic silica production within a time span of 10^4^–10^5^ yr, and (2) a sustained reduction in the sinking flux of radiolarian silica for ~0.3 Myr after the impact. The catastrophic collapse of the pelagic ecosystem at this time was probably the dominant factor responsible for the end-middle Norian conodont extinction.

The biodiversity crisis at the Triassic–Jurassic boundary (TJB) has traditionally been identified as one of the “big five” mass extinctions of the Phanerozoic^1^. Although diversity depletions at the end-Ordovician, end-Permian, and end-Cretaceous intervals resulted exclusively from elevated levels of extinction, the TJB diversity loss was primarily the result of attrition related to reduced origination, along with a higher background extinction rate in the Late Triassic^2^,^3^. Available biostratigraphic data suggest that prominent faunal groups in the marine realm, such as radiolarians, conodonts, and ammonoids, experienced a three-step extinction during the last 15 Myr of the Late Triassic: at the end of the middle Norian, at the end of the Norian, and at the end of the Rhaetian (Fig. 1). Catastrophic processes, such as episodes of anoxia and mantle plume volcanism, in the Central Atlantic Magmatic Province (CAMP) have been proposed to account for the second (end-Norian) and third (end-Rhaetian) extinction events^4^,^5^,^6^. However, the cause of the initial (end-middle Norian) extinction event has been uncertain, despite the fact that it was apparently of global extent.

Here, we show that the remarkable turnover of siliceous plankton (radiolarians) in the end-middle Norian was triggered by a large impact event. Such an event has been inferred from anomalous concentrations of platinum group elements (PGEs)^7^,^8^ and negative Os isotope excursions^9^ in a claystone layer in an Upper Triassic bedded chert succession in the Sakahogi section, Japan (Supplementary Fig. S1), which accumulated in a deep seafloor environment in an equatorial region of the palaeo-Pacific Ocean (Panthalassa)^10^,^11^. The claystone layer is 4–5 cm thick and contains a lower and an upper sublayer (Supplementary Fig. S3). The lower sublayer contains microspherules in a matrix of clay minerals (mainly illite), cryptocrystalline quartz, and hematite. The upper sublayer is composed of undisturbed clay minerals (illite) and cryptocrystalline quartz. The late middle Norian age of the clay layer^7^,^11^ suggests that the PGE anomalies and microspherules in the lower sublayer originated from an extraterrestrial source, related to an impact event that formed the 90-km-diameter Manicouagan crater in Canada (214–215 Ma)^12^,^13^. In addition, a magnetostratigraphic analysis shows a normal polarity interpretation for the clay layer^11^, which is consistent with the palaeomagnetic data for the Manicouagan melt rock^14^. Studies of PGEs and Os isotopes have revealed that the anomalously high PGE abundances in the lower sublayer resulted from a large chondritic impactor with a diameter of 3.3–7.8 km (refs 8 and 9). An impactor of this size would produce a crater of ~56–101 km in diameter^8^, assuming an impactor entry velocity of 20 km s^−1^, an entry angle of 45° and a crystalline target (density = 2750 kg m^−3^). The size range of such a crater is consistent with the size of the Manicouagan crater (diameter, ~90 km)^15^. Outside of Japan, a Norian impact ejecta deposit with shocked quartz and spherules has been found in an Upper Triassic nonmarine sequence in southwestern Great Britain, which has yielded a diagenetic age (from authigenic K-feldspar) of 214 ± 2.5 Ma (ref. 16). Although the recalculated age of 216.7 Ma for the British ejecta deposit using the method of Renne and coworkers^17^,^18^ is slightly outside the range of the Manoucouagan impact event age, a strong mineral correspondence between samples from Manicouagan and the ejecta deposit provides convincing evidence that the Manicouagan impact was the source of the Great Britain ejecta deposit^19^.

## Results

### Extinction and origination patterns

**Figure 2:**
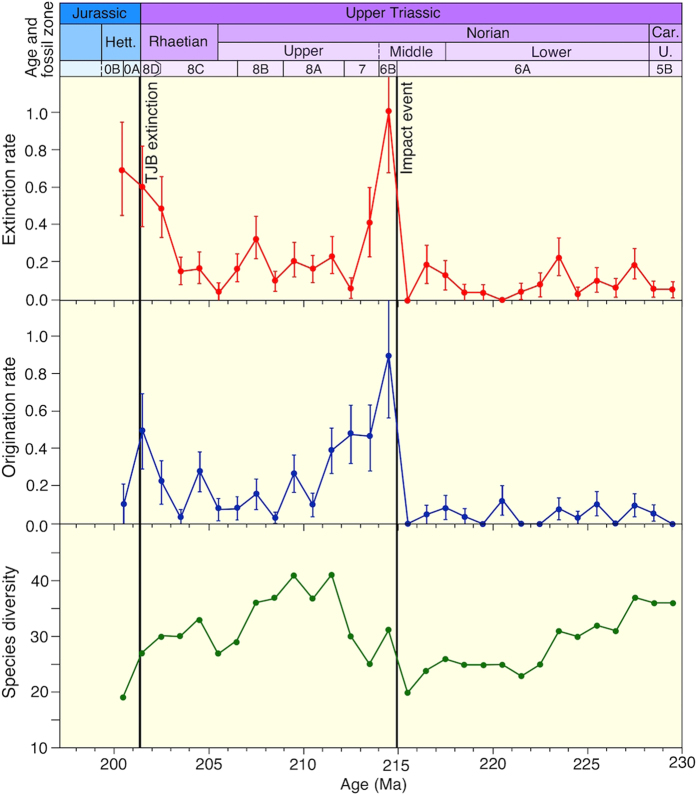
Extinction and origination rates of Late Triassic radiolarian species in the Panthalassa Ocean. The extinction rate of the middle Norian impact event is substantially higher than the rate at the Triassic–Jurassic boundary (TJB). Error bars are one standard deviation, estimated from bootstrap resampling of the stratigraphic ranges of species with 1000 iterations. See Supplementary Fig. S2 for the age model.

We analysed a large dataset of Upper Triassic radiolarian occurrences in the Inuyama area^7^,^20^,^21^,^22^, to assess the magnitude of the extinction event caused by the middle Norian impact, and to compare this event with extinction events at other stage boundaries. An analysis of the stratigraphic ranges of radiolarian species in Japan indicates a dramatic increase in extinction and origination rates across the impact horizon (Fig. 2). Radiolarians show similar (and relatively low) extinction and origination rates throughout much of the late Carnian–middle Norian. Across the ejecta horizon, a dramatic increase in extinction rate is observed in the 1-Myr interval following the impact event. Such a high extinction rate of radiolarians has not been recognized within the Norian interval^23^, and surprisingly, the magnitude of the extinction event is substantially higher than that of the radiolarian extinction at the TJB^21^. Radiolarian species diversity data show a steady decrease in diversity throughout the late Carnian to middle Norian. A sharp increase in diversity is observed across the ejecta horizon, and the diversity reaches a maximum value of 40 species (without singletons) during the late Norian. The diversity then declines rapidly throughout the Rhaetian, reaching a low point in the Hettangian (19 species). These results suggest that the middle Norian impact triggered the extinction and contemporaneous evolutionary radiation of the radiolarian fauna in the equatorial Panthalassa Ocean.

### Biostratigraphy and chemostratigraphy

**Figure 3:**
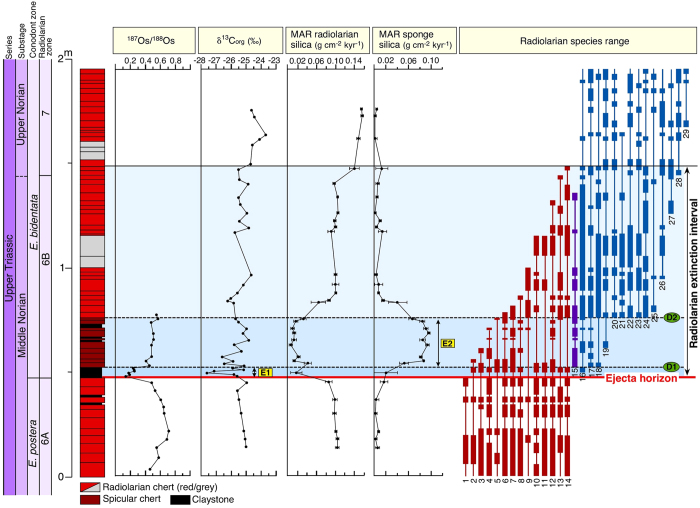
Biostratigraphy and chemostratigraphy of the middle–upper Norian. Stratigraphic profiles of Os isotope ratios^9^, organic carbon isotopes, mass accumulation rates of biogenic silica, and radiolarian biostratigraphy in bedded cherts of the Sakahogi section. Biostratigraphic ranges of 29 radiolarian species in the study interval at Sakahogi show extinctions of middle Norian species (red) corresponding with successive blooms of opportunistic species (purple) and radiations of new species (blue). Dashed lines mark the initial (D1) and second (D2) phases of diversification of upper Norian radiolarian species. For an explanation of radiolarian taxon ranges, see Supplementary Table S4.

An analysis of patterns of radiolarian extinction (Fig. 2) reveals a high extinction rate in the 1 Myr interval after the impact event. To determine whether the extinctions were coincident with the impact event, we conducted a detailed bed-by-bed biostratigraphic analysis of the ~2 Myr interval across the ejecta horizon (Fig. 3). Our biostratigraphic analysis of the study section clearly shows that the impact event initiated the radiolarian extinction and the evolutionary radiation of new taxa. The present data show no catastrophic pattern^24^ of radiolarian extinctions at the impact event horizon. However, gradual extinctions of radiolarians are observed within ~1 m above the ejecta horizon, in the end-middle Norian (Fig. 3). A biostratigraphic analysis shows that 14 middle Norian taxa gradually disappeared during this interval, including all *Capnodoce* and most *Capnuchosphaera* species. An initial phase of diversification (phase D1) started in the upper sublayer of the claystone and in the overlying chert bed. A second major wave of diversification (phase D2) is observed ~33 cm above the ejecta horizon. Given an average sedimentation rate of 1.1 mm kyr^−1^ in the middle Norian chert succession (Supplementary Fig. S2), this second diversification occurred within ~0.3 Myr after the impact event.

To evaluate changes in primary productivity, we examined stratigraphic variations in organic carbon isotopic values (δ^13^C_org_) from the study section at Sakahogi (Supplementary Table S1). The data show that δ^13^C_org_ values are relatively constant below the ejecta claystone (average δ^13^C_org_, −25.22‰), but that a negative δ^13^C_org_ excursion is present in the upper claystone sublayer (Fig. 3). The δ^13^C_org_ values increase rapidly upwards, and then recover to pre-impact levels at the first chert bed overlying the claystone layer (−25.13‰). Above the claystone interval, δ^13^C_org_ values vary from −27.09‰ to −24.57‰ (average δ^13^C_org_, −25.44‰).

## Discussion

A preliminary biostratigraphic study of the section at Sakahogi shows that most of the middle Norian radiolarian taxa are present in the 30 cm interval above the ejecta layer, and apparently survived well into the late Norian^7^ (Supplementary Fig. S4). However, our new biostratigraphic data collected from above the previously sampled horizon indicate that extinctions of middle Norian species occurred in a stepwise fashion in the ~1 m interval above the ejecta horizon. Furthermore, our high-resolution palaeontological and geochemical data reveal that two palaeoenvironmental events occurred during the initial phase of the radiolarian extinction interval (Fig. 3). The first event (E1) consisted of the post-impact shutdown of primary productivity and a remarkable decline in the amount of biogenic silica preserved before the first phase of diversification (D1). The second event (E2) consisted of a large and sustained reduction in the sinking flux of radiolarian silica and the proliferation of siliceous sponges, occurring before the second phase of diversification (D2) and lasting for ~0.3 Myr after the impact.

During the initial E1 event, the post-impact reduction in primary productivity occurred during deposition of the clay layer, as suggested by the negative δ^13^C_org_ excursion observed in the claystone. Concentrations of TOC with low δ^13^C_org_ values could be interpreted to represent a change in the proportion of organic matter derived from land plants versus oceans^25^,^26^. However, low and relatively stable C/N ratios (mean, 1.8; standard deviation, 0.6) throughout the study section indicate that contributions of land-derived organic matter are minor to absent. The rapid decrease in the biogenic silica content in the claystone (Supplementary Table S3) further indicates that the productivity of silica-secreting radiolarians was sharply reduced, and that marine primary productivity declined during the initial E1 event. Laboratory studies show that radiolarians prey on phytoplankton, such as dinoflagellates, haptophytes, and thecate nonmotile algae^27^,^28^; however, we know little about the ecological factors that influence radiolarian productivity. A decline in radiolarian production in the near-surface zone may imply major changes at the base of the marine food chain, such as a substantial reduction in primary productivity or a shift toward primary producers not favored by pre-impact taxa.

The negative δ^13^C_org_ excursion and the rapid decrease in biogenic SiO_2_ content observed in the upper part of the claystone occurs within a 3.2-cm-thick section, suggesting a very short time scale for the E1 event. We used previously published ^187^Os/^188^Os ratio results^9^ to estimate the duration of low-productivity conditions after the impact. Previous studies have revealed that ^187^Os/^188^Os ratios declined abruptly, from 0.477 to 0.126, in the lower sublayer of the claystone. This excursion can be interpreted as a result of the mixing of ambient seawater Os (characterized by relatively high ^187^Os/^188^Os ratios) with meteoritic Os (characterized by low ^187^Os/^188^Os ratios); the meteoric Os was vaporized at the time of the impact and was subsequently dissolved into seawater. The ^187^Os/^188^Os ratio, which is lowest in the lower sublayer, gradually increases towards the upper sublayer, and reaches pre-impact levels in chert samples overlying the claystone layer. No discrete extraterrestrial particles were observed in the upper sublayer claystone, which indicates that the ^187^Os/^188^Os ratio of this sublayer records the Os isotope composition of ancient seawater^9^. Therefore, the recovery of ^187^Os/^188^Os values in the upper sublayer claystone after the impact event may reflect post-impact removal of excess dissolved meteoritic Os from seawater, which occurred over a period of 10^4^–10^5^ yr, as Os residence times range from 10 to 60 kyr (refs 29 and 30). Although the marine residence time of Os in the Late Triassic is not precisely known, deposition of the upper sublayer clay may have occurred over the period 10^4^–10^5^ yr after the impact event. This interval is hypothesized to represent the duration required for the restoration of productivity by primary and silica-secreting organisms after the middle Norian impact. However, simulations using an ocean–atmosphere/carbon-cycle model^31^, which suggest a global collapse of primary productivity (in the Strangelove Ocean) resulting in the delivery and cycling of carbon in the oceans and on land, cannot explain such short-term (10^4^–10^5^ yr) shifts in δ^13^C_org_ values across the ejecta layer. The global implications of the magnitude and short duration of the negative δ^13^C_org_ excursion reported here remain to be verified at other middle–late Norian boundary intervals worldwide.

Following the resurgence in primary productivity after the E1 event, the biogenic silica content had recovered to pre-impact values by the first chert bed overlying the claystone layer. However, our analysis reveals that major biotic components of the bedded chert changed temporarily from radiolarians to siliceous sponges, for ~0.3 Myr after the impact. Assuming a constant sedimentation rate in the middle–upper Norian chert succession of 1.1–1.6 mm kyr^−1^, and a constant dissolution rate of biogenic silica during that time, the mass accumulation rates (MAR) of radiolarian silica in the pre-impact chert beds is estimated at ~0.1 g cm^−2^ kyr^−1^, which is the same as the biogenic silica flux near the equator in the modern Pacific Ocean (0.1–0.3 g cm^−2^ kyr^−1^)^32^. After the impact event, the MAR decreased to 0.02 ± 0.01 g cm^−2^ kyr^−1^ in the upper sublayer claystone, and then remained low during the deposition of sponge spicule-rich cherts. On the other hand, the MAR of the siliceous sponge silica increased markedly across the claystone layer of the ejecta deposit, from ~0.01 to ~0.08 g cm^−2^ kyr^−1^, and subsequently decreased to ~0.01 g cm^−2^ kyr^−1^ after the E2 event. Our data and previous data^9^ on terrigenous elements (e.g., Ti, Al, and K) indicate that the flux of terrestrial components (aeolian dust) derived from continental crust^22^,^33^ did not change substantially through the study interval; thus, the significant and sustained reduction in the flux of radiolarian silica appears to have coincided with an increasing volume of siliceous sponge spicules. During the Mesozoic, the concentration gradient caused by the export of silica from surface to deep waters by sinking of marine plankton may not have been as intense as in modern diatom-dominated oceans^34^, and such concentration gradients in any case would have been briefly interrupted by fluctuations in radiolarian productivity. Even lacking a direct record of Triassic silica concentrations, it is likely that a reduction in radiolarian productivity in the Panthalassa Ocean during the E2 event, which probably increased the amount of dissolved silicic acid in seawater, favored the proliferation of siliceous sponges after the impact event. Laboratory experiments on silicon uptake by siliceous sponges reveal that an increased concentration of silicic acid in water has a striking positive effect on both the size and robustness of siliceous sponge spicules^35^. Our hypothesis that dissolved silicic acid increased in seawater at the time of the E2 event is supported by the observation that longer and more robust skeletons of siliceous sponge spicules were dominant only in the spicules of the spicule-rich chert (Supplementary Fig. S10).

Decreases in the sinking flux of radiolarian silica during the E1 and E2 events may reflect a decline in radiolarian production in middle Norian taxa, including in *Capnodoce* and *Capnuchosphaera* species. These middle Norian radiolarians are very rare above the E1 interval, whereas a small spumellarian species is abundant within the E2 interval; this spumellarian species is reported as Spumellaria gen. et sp. indet. A, and its occurrence can be used to identify the stratigraphic position of the ejecta layer in other Triassic chert sections within the Jurassic accretionary complexes in Japan^9^. These taxa can be considered as short-lived opportunistic species, as they disappeared at the end of the radiolarian faunal turnover interval. The present biostratigraphic analysis also reveals that radiation of late Norian taxa was contemporaneous with a temporal bloom in the numbers of opportunistic spumellarian species in the E2 interval. The timing of these radiation events suggests that the decrease in radiolarian biomass in the middle Norian taxa enhanced the bloom of opportunistic radiolarian species and the evolutionary radiation of late Norian taxa in the E1 and E2 intervals. Hence, the gradual extinction of middle Norian radiolarian taxa during the ~1 Myr period could be explained by ecological pressures imposed by late Norian taxa, provided that the late Norian taxa were more rapidly growing and more efficient phytoplankton feeders than the middle Norian taxa. These unusual radiation patterns are similar to those observed in the Panthalassic TJB sections in Japan and Canada^21^,^22^. As with the TJB event, changes in seawater acidity, temperature, and/or a reduced nutrient levels in ocean surface waters are possible drivers for the decline in the production of middle Norian radiolarian taxa. The primary cause of this decline is difficult to identify, but the relatively long period of the E2 interval (~0.3 Myr after the impact) largely excludes the possibility that the decline was triggered by instantaneous environmental stresses (e.g., extended darkness, global cooling, or acid rain^24^,^36^) that would have been caused by a bolide impact.

Did the middle–late Norian extinction event occur uniformly on a global scale, or does it represent a regional phenomenon in the Panthalassa Ocean? The record of radiolarian faunal change across the middle–upper Norian boundary has been established at the species level in several regions^37^,^38^, showing that widespread and apparently sudden extinctions affected the Subfamily Capnodocinae and Family Capnuchosphaeridae at the boundary^23^. As a first approximation, it is probably reasonable to assume that a geographically widespread faunal change across the middle–upper Norian boundary was related to an impact event that triggered the radiolarian extinction in the equatorial Panthalassan Ocean. Existing radiolarian records are not sufficiently precise to constrain these relationships with biostratigraphic resolutions comparable to those presented here for the middle–upper Norian. Further biostratigraphic analyses of middle–upper Norian boundary sections will be required to validate this hypothesis.

We propose that the impact event was probably the major factor responsible for the conodont and Pacific (North American) ammonoid extinctions that occurred in the middle–upper Norian boundary^39^,^40^,^41^. The base of the *Epigondolella bidentata* conodont zone and the base of the *Gnomohalorites cordilleranus* ammonoid zone in western North America^39^,^40^ define the position of the middle–upper Norian boundary that is most closely aligned with the traditional base of the Sevatian^41^, and which can be correlated with the radiolarian extinction interval in the study section in Japan. Although ammonoids are absent in the studied section, the biostratigraphic record of conodonts suggests that a few *Parvigondolella* species survived across the ejecta layer, but that an important middle Norian *Epigondolella* species became extinct just below the impact horizon (Supplementary Fig. S11). The present data also show the first appearance of late Norian *Epigondolella* species in the E1 and E2 intervals. Conspicuous morphological changes occur in this genus across the ejecta layer; *Epigondolella* species below the ejecta layer are characterized by a wide platform, whereas those above the ejecta layer possess a longer and more narrow platform. The catastrophic collapse of the pelagic ecosystem during the E1 and E2 events was probably the major factor responsible for the conodont turnover that occurred at the end of the middle Norian.

This study has revealed that late middle Norian open-ocean ecosystems experienced profound disruptions after a large impact event (chondritic impactor of 3.3–7.8 km in diameter), and that the event was possibly related to the 90-km-diameter Manicouagan crater in Canada. Although many marine sections over the past 540 Myr have been examined, no catastrophic collapse in marine ecosystems caused by an extraterrestrial impact has yet been described, with the outstanding exception of the Cretaceous–Paleogene boundary (KPB) crisis^24^ and the middle Norian event reported here. Given that no large volcanic events occurred in the Norian^3^, the fossil record of the middle–upper Norian is key to evaluating the general importance of impacts as causes of biotic and environmental changes in pelagic ecosystems.

## Methods

### Biostratigraphy

To extract radiolarians for biostratigraphic analyses, 53 chert samples were soaked in a dilute HF solution (5%) for 24 h, and were then passed through a 32-μm mesh sieve. This treatment was repeated 2–5 times to obtain sufficient residue for analysis. After drying the residue, radiolarians were handpicked under a binocular microscope and were observed using a scanning electron microscope. Conodonts were handpicked from the same residues used for the radiolarian biostratigraphy.

### Carbon isotope and major element analyses

Samples for whole-rock geochemical analysis were collected from 45 chert and claystone beds across the ejecta layer. Veins and strongly recrystallized/weathered parts of the samples were avoided to minimize the effects of diagenetic and metamorphic overprinting on the sediment geochemistry. Samples were crushed and fragments were carefully hand-picked to avoid contamination by altered and weathered materials. These hand-picked fragments were then pulverized in an agate mortar for carbon isotope analysis. All glassware used in the analyses was baked for 3 h at 450 °C, and all tools were cleaned with methanol between uses to avoid organic carbon contamination. Approximately 0.8 g of each sample was treated with an excess of 6 M hydrochloric acid for 48 h at 60 °C. Samples were then rinsed five times with ultrapure water (>18 MΩ) and dried. Carbon isotope ratios and carbon and nitrogen contents were measured using an elemental analyser (FlashEA 1112) coupled to a Thermo-Finnigan Delta Plus Advantage isotope ratio mass spectrometer at the Centre for Advanced Marine Core Research, Kochi University, Japan. Isotopic measurements were repeated two times for 19 samples across the claystone (ejecta) layer to check the reproducibility of the results. The carbon isotope ratios were calculated using standard Alanine; the precision of the organic carbon ratio (δ^13^C_org_) determinations was ± 0.5‰. The analyses showed good reproducibility of values, with 3% or less fluctuation (average, 2%; N = 18). All isotopic results are reported in conventional delta (δ) notation, defined as per mill (‰) deviations from the Vienna Peedee Belemnite (VPDB) standard value.

Major element (Si, Ti, Al, Fe, Mn, Mg, Ca, Na, K, and P) contents of the bulk-sediment samples from the cherts and claystones in the Sakahogi section were measured using a Rigaku ZSX Primus II X-ray fluorescence (XRF) spectrometer at the University of Tokyo. After drying the powdered samples at 110 °C for ~12 h, loss on ignition (LOI) was calculated from the weight loss during ignition at 950 °C for over 6 h. Fused glass beads for XRF analysis were made from a mixture of 0.4 g of ignited sample powder and 4 g of lithium tetraborate (Li_2_B_4_O_7_) flux at ~1190 °C for 7 min in a Pt crucible. The analytical results were generally within 3% (relative percent difference) of accepted values of reference material JB-2, issued by the Geological Survey of Japan^42^.

### Diversity measurements

Regional species-level diversities of radiolarians throughout the Carnian to Hettangian were measured in 10 chert sequences in the Inuyama area, Japan. The data comprise 2196 occurrences of 156 radiolarian species from 361 stratigraphic levels in the study sections; the collection intensity was uniform and without sampling gaps (Supplementary Fig. S2). Species ranges were analysed using metrics established by Foote^43^, including: total diversity, total diversity minus singletons, estimated mean standing diversity, per-taxon rate, and the Van Valen rate. Extinction (E) and origination (O) rates reported here were calculated for 1-Myr intervals using Foote’s per-taxon rate:


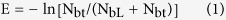



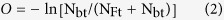


where N_bt_ is the number of taxa crossing both the bottom and top of an interval, N_bL_ is the number of taxa crossing the bottom but not the top of the interval, and N_Ft_ is the number of taxa first appearing in the interval and crossing the top of the interval. Errors for the extinction and origination rates are represented by one standard deviation determined in each direction of a bootstrap resampling of the stratigraphic ranges of species based on 1000 iterations. To estimate per-taxon rates for the 1-Myr intervals, we constructed an age model for the Upper Triassic bedded chert sequence based on the average sedimentation rate estimated from the measured thickness and the time interval of its deposition. Extinction and origination patterns of radiolarian species show that the derived rates for all four metrics used (i.e. per-taxon rate, per-taxon rate without singletons, Van Valen with singletons, and Van Valen without singletons) have similar values. Consequently, only the per-taxon rate with singletons is discussed.

### Productivity reconstruction

The Triassic bedded cherts in the Inuyama area comprise biogenic silica (radiolarian tests and siliceous sponge spicules) and terrestrial components derived from the continental crust (aeolian dust)^22^,^33^. Biogenic silica (BSi) was calculated as follows:


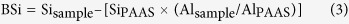


where PAAS is a shale standard based on the composition of post-Archean average Australian shales^44^. In this calculation, the amount of terrigenous Si that is mainly supplied by aeolian dusts is subtracted from the total concentration of Si in the bedded cherts. To assess the relative contributions of the radiolarian and sponge silica to the BSi, we determined the volumetric composition of radiolarians and siliceous sponges three times by standard 300-grain points counts (grain-solid method) for 118 thin sections. The percentages of radiolarian and sponge silica (Si_rad_ and Si_sponge_, respectively) were then calculated as follows:


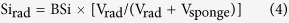



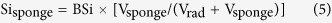


where V_rad_ and V_sponge_ are the volumetric compositions of the radiolarians and siliceous sponges based on thin section analysis, respectively. Then, the mass accumulation rate (g cm^−2^ kyr^−1^) of radiolarian and sponge silica (MAR_rad_ and MAR_sponge_, respectively) was calculated as follows:


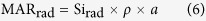



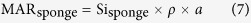


where *ρ* is the bulk density of chert (2.46 g cm^−3^) or claystone (1.69 g cm^−3^) and *a* is the sedimentation rate for chert (0.11–0.16 cm kyr^−1^) or claystone (0.09 cm kyr^−1^). The sedimentation rate of the claystone layer was calculated from the measured thickness (3.2 cm), and the time interval of its deposition was estimated using a mean oceanic residence time of Os (~3.5 kyr)^29^, as discussed above.

## Additional Information

**How to cite this article**: Onoue, T. *et al*. Bolide impact triggered the Late Triassic extinction event in equatorial Panthalassa. *Sci. Rep.* **6**, 29609; doi: 10.1038/srep29609 (2016).

## Supplementary Material

Supplementary Information

## Figures and Tables

**Figure 1:**
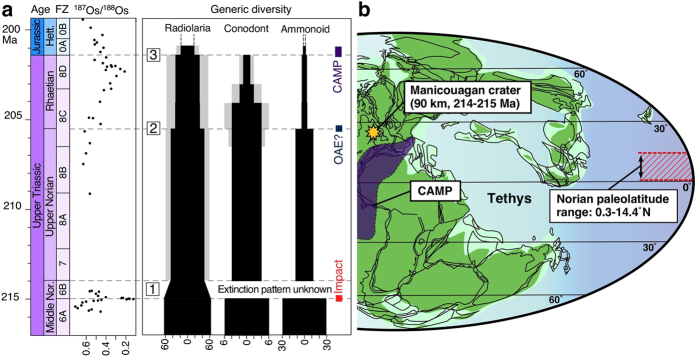
Osmium isotope values, generic diversity, and palaeogeography in the Late Triassic. (**a**) Late Triassic generic diversities of radiolarians^23^, conodonts^41^, and Pacific (North American) ammonoids^40^, as compared with the Os isotope record^9^,^45^ in the Panthalassa Ocean. The abrupt decrease in the ^187^Os/^188^Os ratio in the middle Norian is synchronous with the Manicouagan impact event at 214–215 Ma. Stepwise or episodic extinctions in the (1) end-middle Norian, (2) end-Norian, and (3) end-Triassic are possibly linked with a large bolide impact^7^, an oceanic anoxic event (OAE)^4^, and the Central Atlantic Magmatic Province (CAMP) volcanic event^5^,^6^, respectively. The gradual decrease in radiolarian diversity just prior to the end-middle Norian may have occurred within radiolarian biozone 6B. Gray shaded areas in the radiolarian and conodont generic diversities represent the number of genera; the genera first appear in the upper Norian and Rhaetian. The geological time scale is from refs 46 and 47. Triassic radiolarian fossil zones (FZ) and their age correlations are from refs 7, 20 and 48; the biostratigraphic framework for our age model is shown in Supplementary Fig. S2. (**b**) Late Triassic palaeogeographic map showing approximate locations of the Manicouagan carter and the inferred depositional area of the bedded chert in the Mino Belt, in low-latitude zones of the Panthalassa Ocean^11^. The map is created using ACD Systems Canvas Draw software (Version 2.0).
