# Supplementary material for: Bolide impact triggered the Late Triassic extinction event in equatorial Panthalassa
Source: Sci Rep. 2016 Jul 8;6:29609. doi: 10.1038/srep29609 (PMC4937377; doi:10.1038/srep29609)
Supplement: Supplementary Information [file srep29609-s1.doc]

**Bolide impact triggered the Late Triassic extinction event in equatorial Panthalassa**

Tetsuji Onoue^1^, Honami Sato^2^, Daisuke Yamashita^1^, Minoru Ikehara^3^, Kazutaka Yasukawa^2,4^, Koichiro Fujinaga^2,5^, Yasuhiro Kato^2,4,5^ and Atsushi Matsuoka^6^

^1^Department of Earth and Environmental Sciences, Kumamoto University, 2-39-1 Kurokami, Kumamoto 860-8555, Japan

^2^Japan Agency for Marine-Earth Science and Technology (JAMSTEC), 2-15 Natsushima-cho, Yokosuka 237-0061, Japan

^3^Centre for Advanced Marine Core Research, Kochi University, B200 Monobe, Nankoku 783-8502, Japan

^4^Department of Systems Innovation, School of Engineering, The University of Tokyo, 7-3-1 Hongo, Bunkyo-ku, Tokyo 113-8656, Japan

^5^Frontier Research Centre for Energy and Resources (FRCER), School of Engineering, The University of Tokyo, 7-3-1 Hongo, Bunkyo-ku, Tokyo 113-8656, Japan

^6^Department of Geology, Niigata University, Igarashi 2-no-cho 8050, Niigata 950-2181, Japan

**Supplementary Information**

**1. Supplementary Figures**


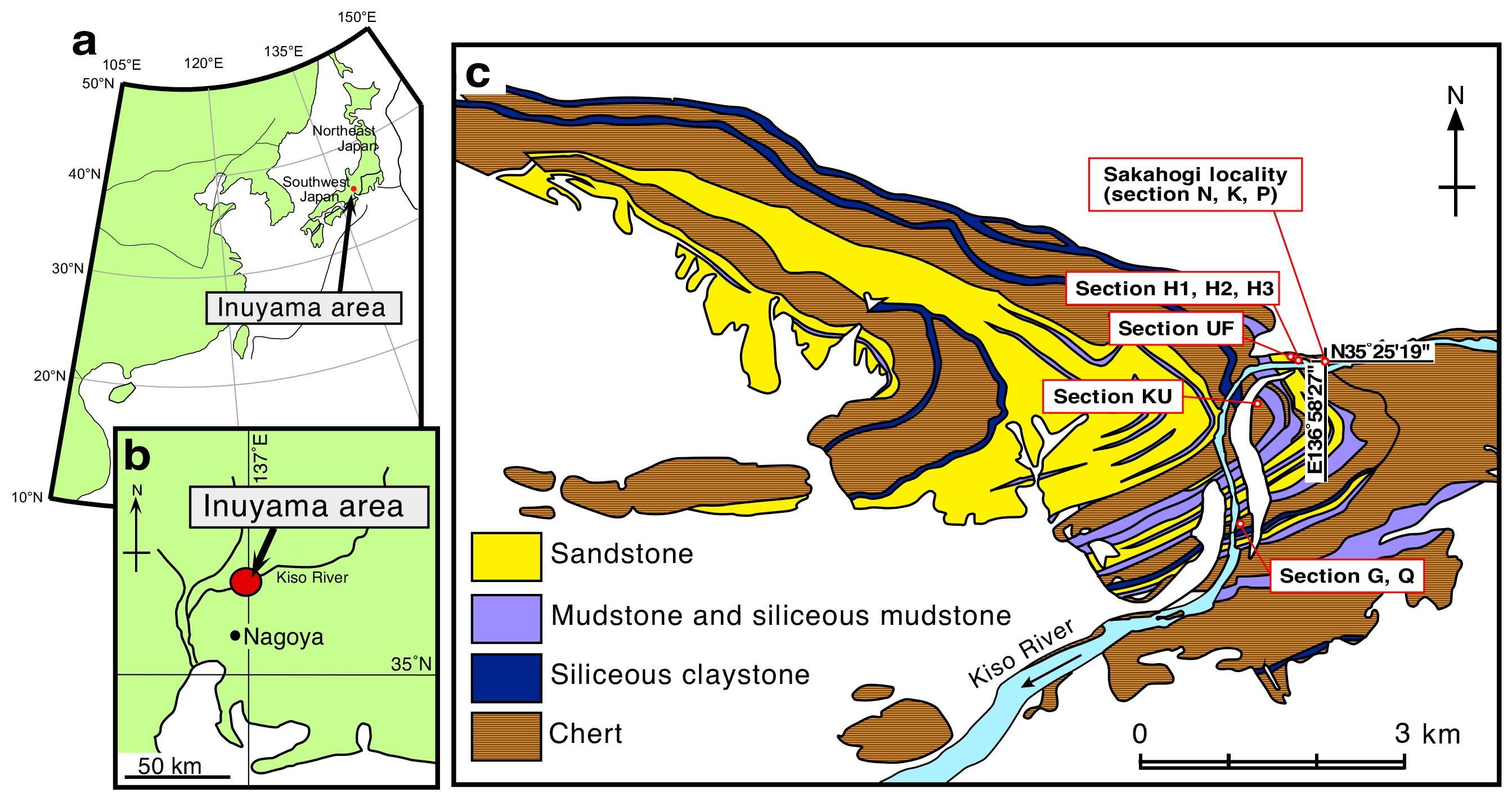


**Supplementary Figure S1. a, b,** Map showing the location of study area in central Japan. **c,** Location of Sakahogi (35°25′19.4″N, 136°58′26.7″E) along the middle reaches of the Kiso River, indicated on a geologic map. The ejecta deposit was discovered in the radiolarian chert succession of the Kamiaso Unit in the Mino Belt, defined as a Jurassic subduction-generated accretionary complex in central Japan. The map is created using ACD Systems Canvas Draw software (Version 2.0).

**
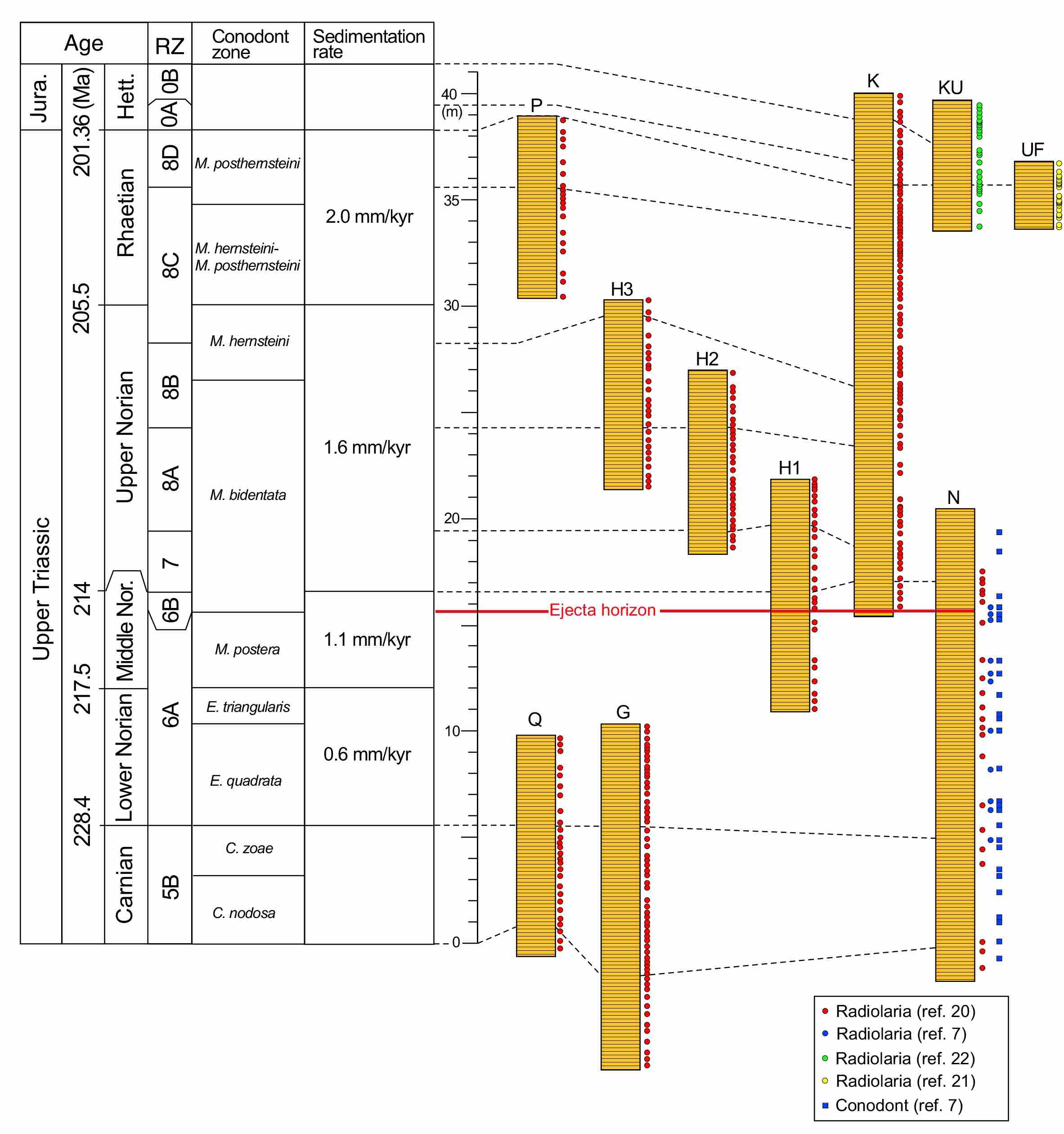
**

**Supplementary Figure S2.** Time-stratigraphic summary of Upper Triassic bedded chert successions in the Inuyama area, Japan. Locations of columnar sections are shown in Supplementary Fig. S1. Triassic radiolarian fossil zones^20^ (RZ) are correlated with conodont zonal schemes and magnetostratigraphic data from the same chert successions^7,11,45^. The radiolarian time scale is further correlated with the radiolarian zonal schemes for the Tethys and western North America regions^48^, and is calibrated to ammonite and conodont zonations. The geological time scales of ref. 46 and ref. 47 are utilized for comparisons of biostratigraphic and chronostratigraphic data. Recent age determinations indicate that the base of the Rhaetian is at 205.5 Ma (ref. 47) rather than the previously determined age of 209.5 Ma (ref. 46).

**Supplementary Figure S3.** Photograph showing detail of claystone layer at Sakahogi locality, modified after ref. 7. The claystone layer is 4–5 cm thick and contains a lower and an upper sublayer. The lower sublayer contains microspherules in a matrix of clay minerals (mainly illite), cryptocrystalline quartz, and hematite. The upper sublayer is composed of undisturbed clay minerals (illite) and cryptocrystalline quartz.


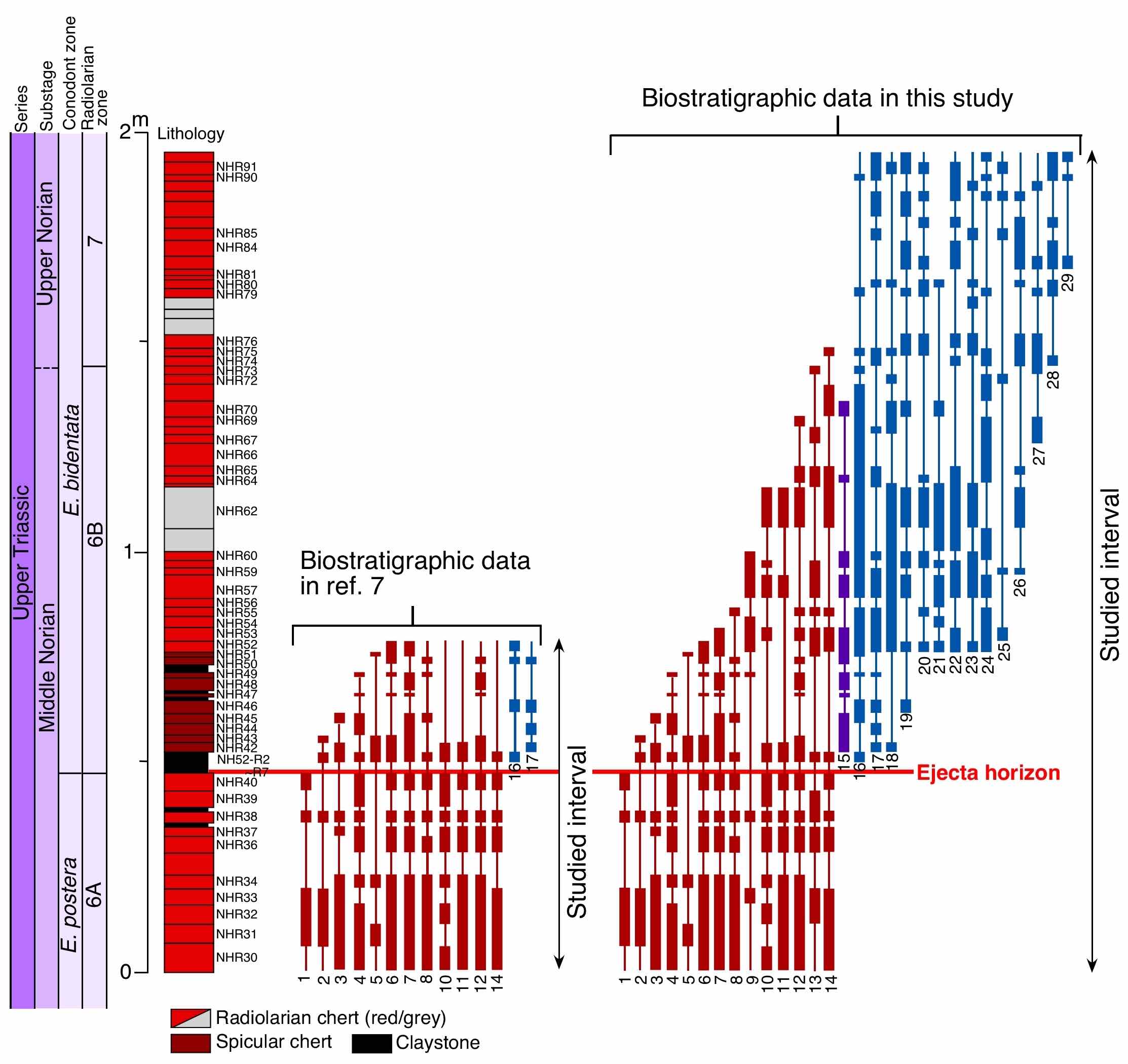


**Supplementary Figure S4.** Comparison of biostratigraphic data from previous studies^7^ and the present study, for the interval at Sakahogi. Our new biostratigraphic data show that extinctions of middle Norian species (red) occurred in a stepwise fashion within a ~1 m interval above the ejecta horizon, coinciding with successive blooms of opportunistic species (purple) and radiations of new species (blue). For an explanation of the ranges of radiolarian taxa, see Supplementary Table S4.


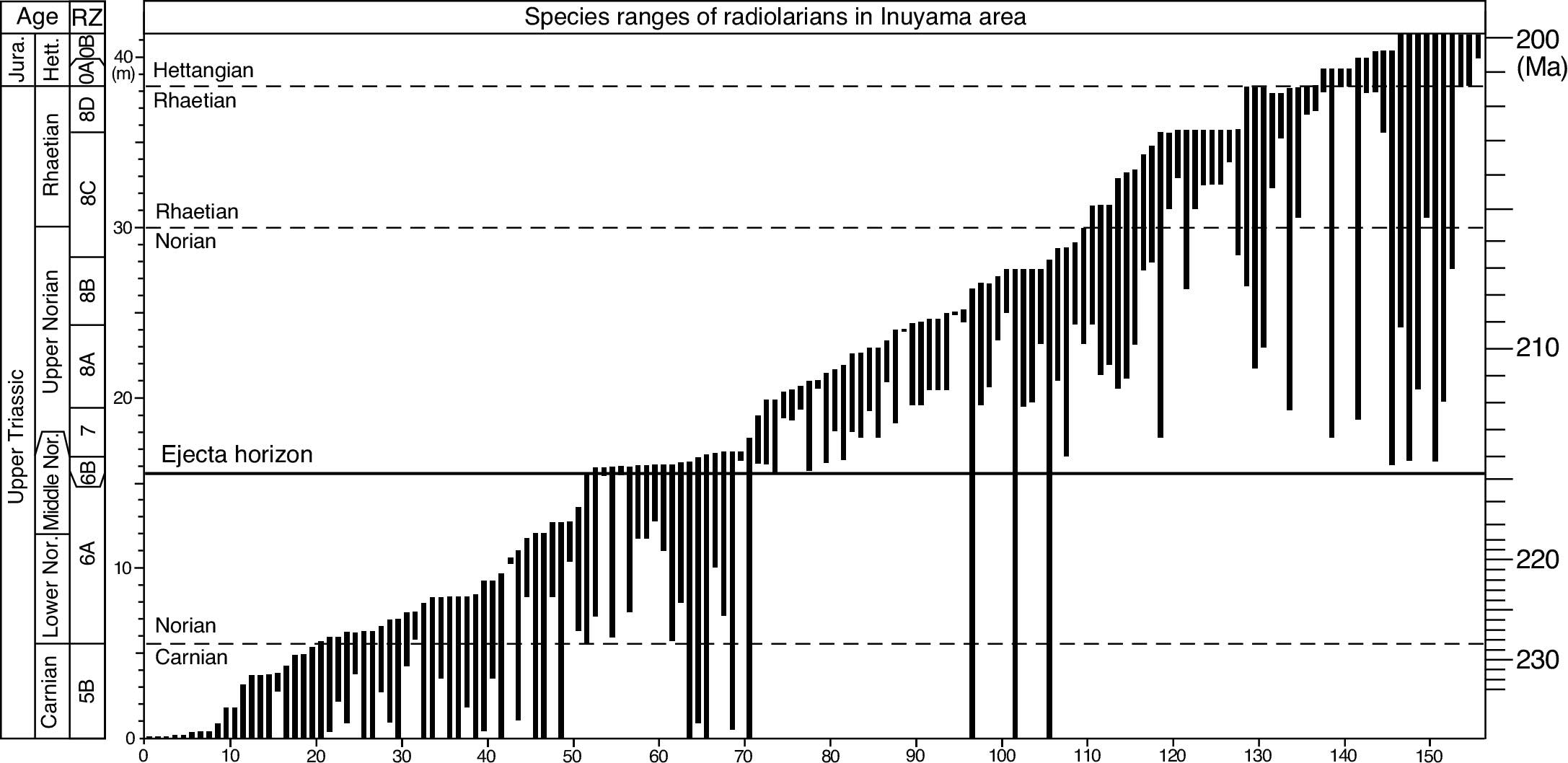


**Supplementary Figure S5.** Stratigraphic ranges of Late Triassic radiolarian species in the Inuyama area, projected onto a composite section, modified after ref. 7. Species numbers are shown on the x-axis. For an explanation of radiolarian taxon ranges, see Supplementary Table S5. Radiolarian zones (RZ) are from ref. 20.


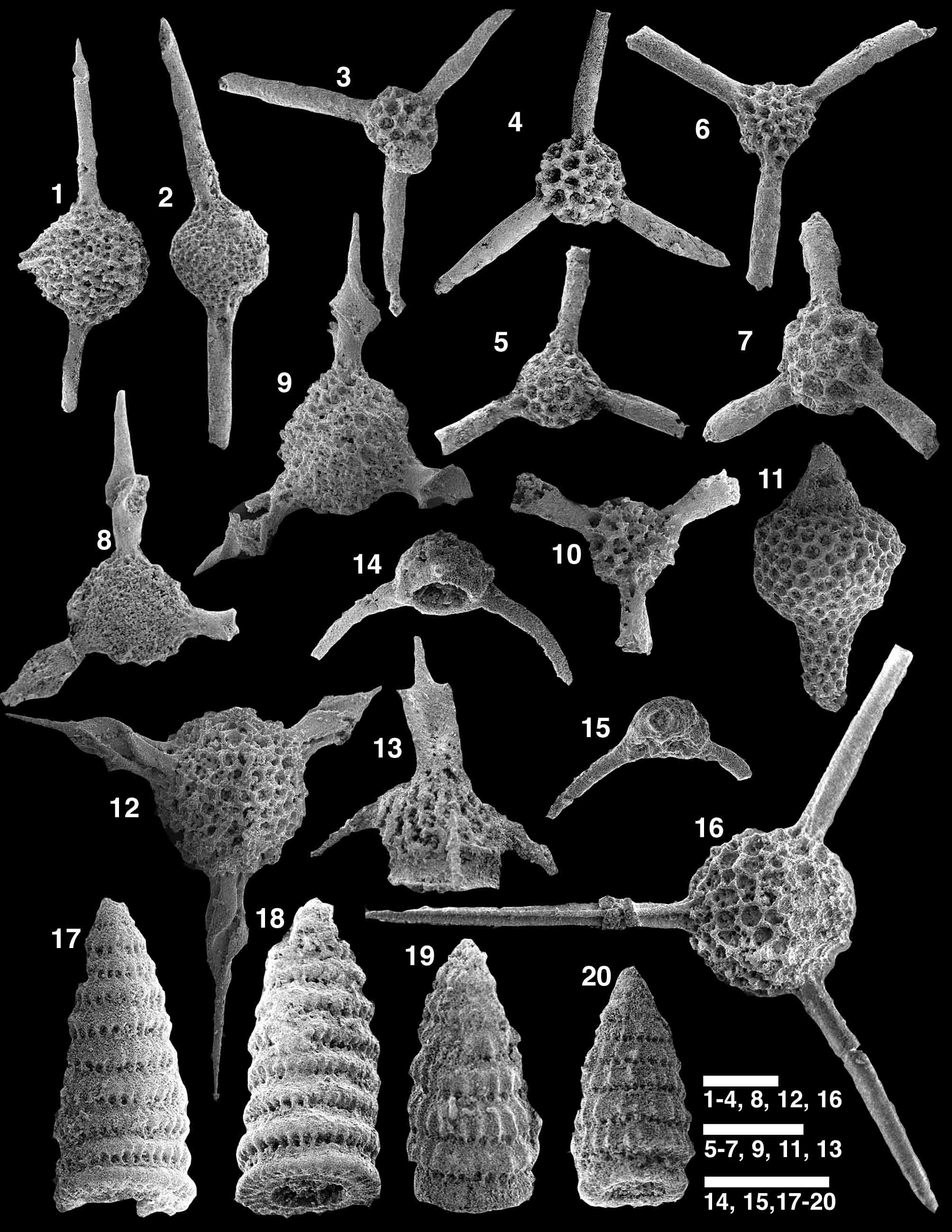


**Supplementary Figure S6.** Middle Norian radiolarians from the study section. Scale bars = 100 µm. **1, 2,** *Xiphosphaera fistulata* Carter. NHR30 (1); NHR49 (2). **3, 4,** *Capnodoce sarisa* De Wever. NH52-R6. **5,** *Capnodoce extenta* Blome. NHR50. **6,** *Capnodoce* sp. cf. *C. ruesti* Kozur and Mock. NH52-R6. **7,** *Capnodoce crystallina* Pessagno. NHR43. **8, 9,** *Capnuchosphaera* sp. cf. *C. deweveri* Kozur and Mostler. NHR36 (8); NHR53 (9). **10,** *Capnodoce* sp. cf. *C. anapetes* De Wever. NHR31. **11,** *Syringocapsa batodes* De Wever. NH52-R6. **12,** *Sarla hadrecaena* (De Wever). NHR57. **13,** *Trialatus robustus* (Nakaseko and Nishimura). NHR33. **14, 15,** *Poulpus piabyx* De Wever. NHR37 (14); NH52-R6 (15**)**. **16,** *Sepsagon longispinosus* (Kozur and Mostler). NHR42. **17, 18,** *Japonocampe nova* (Yao). NHR36 (17); NH52-R6 (18). **19, 20,** *Corum regium* Blome. NHR43 (19); NH52-R6 (20).

**
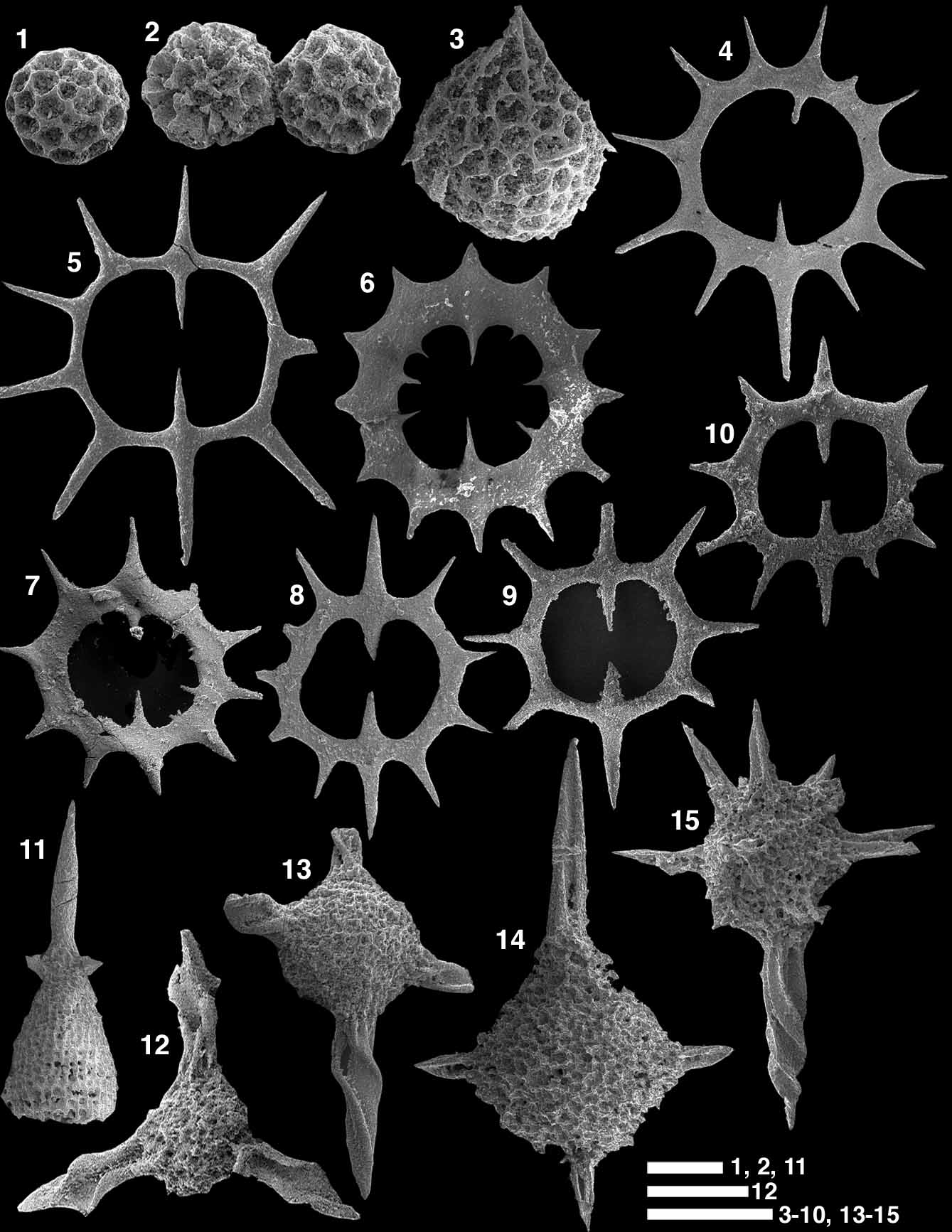
**

**Supplementary Figure S7.** Upper Norian radiolarians from the study section. Scale bars = 100 µm. **1, 2,** Spumellaria gen. et sp. indet. A. NHR42. **3,** *Pentactinocarpus sevaticus* Kozur and Mostler. NHR42. **4,** *Palaeosaturnalis harrisonensis* (Blome). NHR52. **5,** *Palaeosaturnalis* sp. aff. *P. dotti* (Blome). NHR81. **6,** *Pseudoheliodiscus heisseli* (Kozur and Mostler) NHR91. **7,** *Pseudoheliodiscus finchi* Pessagno. NHR62. **8,** *Palaeosaturnalis* sp. aff. *P. harrisonensis* (Blome). NHR60. **9,** *Palaeosaturnalis largus* (Blome) NHR57. **10,** *Palaeosaturnalis dotti* (Blome) NHR57. **11,** *Lysemelas olbia* Sugiyama. NHR80. **12,** *Sarla prietoensis* Pessagno. NHR57. **13,** *Plafkerium* sp. A. NHR60. **14,** *Plafkerium* (?) sp. B. NHR74. **15,** *Discofulmen* sp. NHR90.

**Supplementary Figure S8.** TOC vs δ^13^C_org_ and C/N vs δ^13^C_org_ diagrams for samples from the Sakahogi section.

**Supplementary Figure S9.** Photomicrographs showing spicular (a) and radiolarian (b) cherts from the Sakahogi section. Plane polarized light. NHR 46 (a); NHR 38 (b). Scale bars = 1 mm.


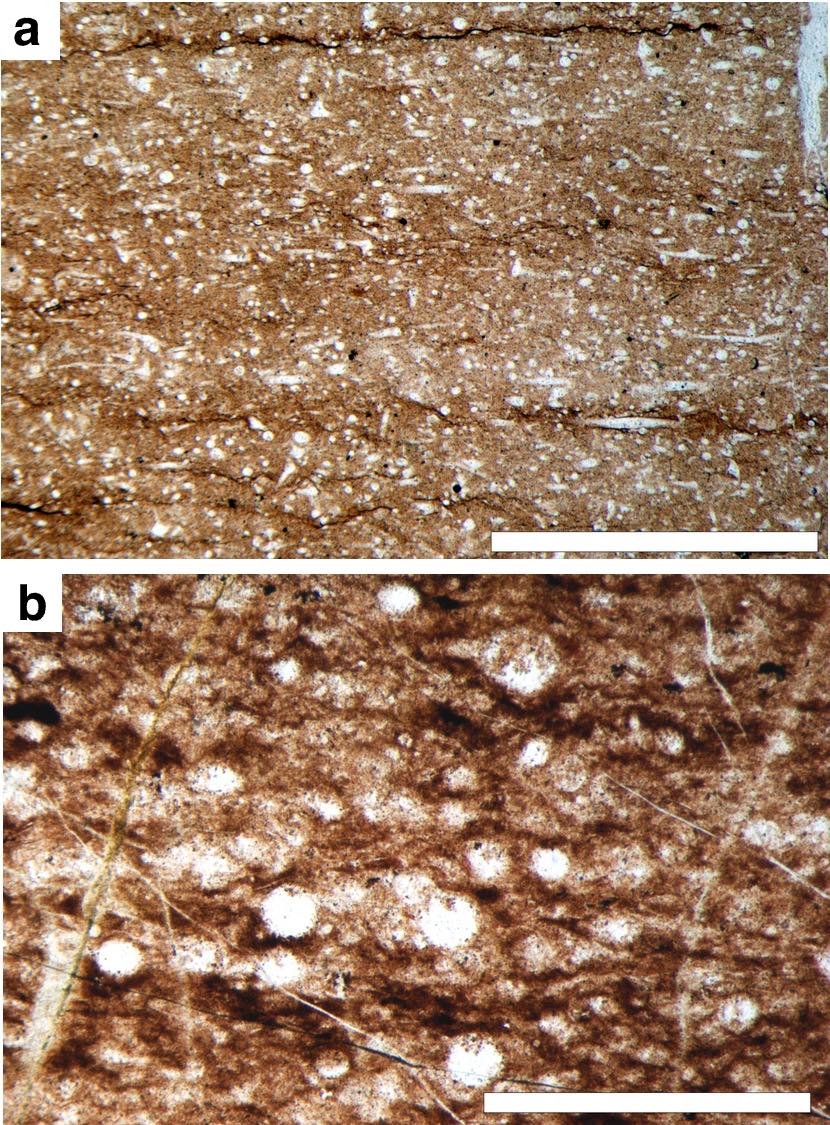


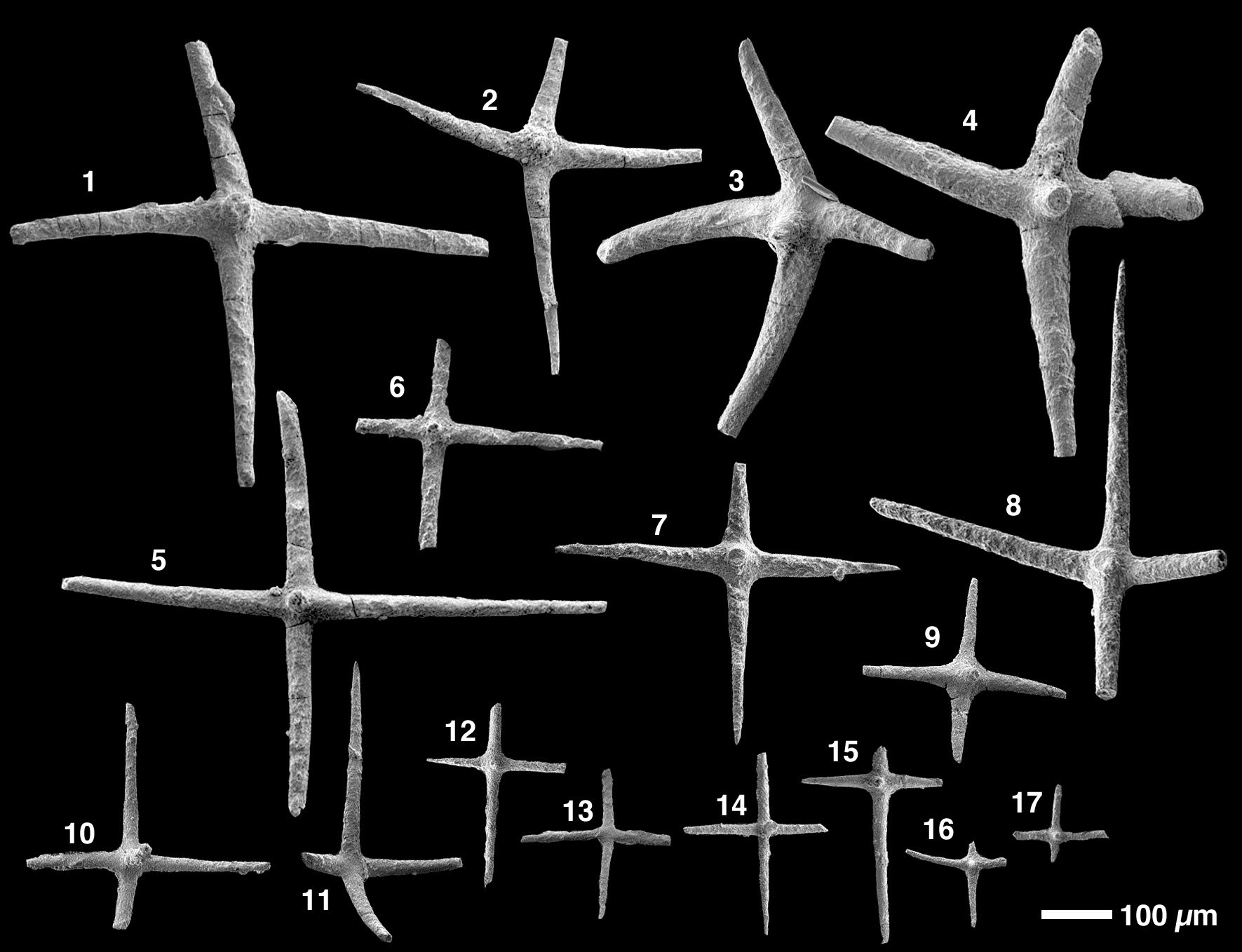
 **Supplementary Figure S10.** Scanning electron microscope (SEM) images showing tetraradiate sponge spicules in a chert sample from the Sakahogi section. The longer and more robust skeletons of sponge spicules (1–9) were dominant above the ejecta horizon. 1–8, NHR49; 9, NHR46; 10–13, NHR32; 14–16, NHR37; 17, NHR40.

**
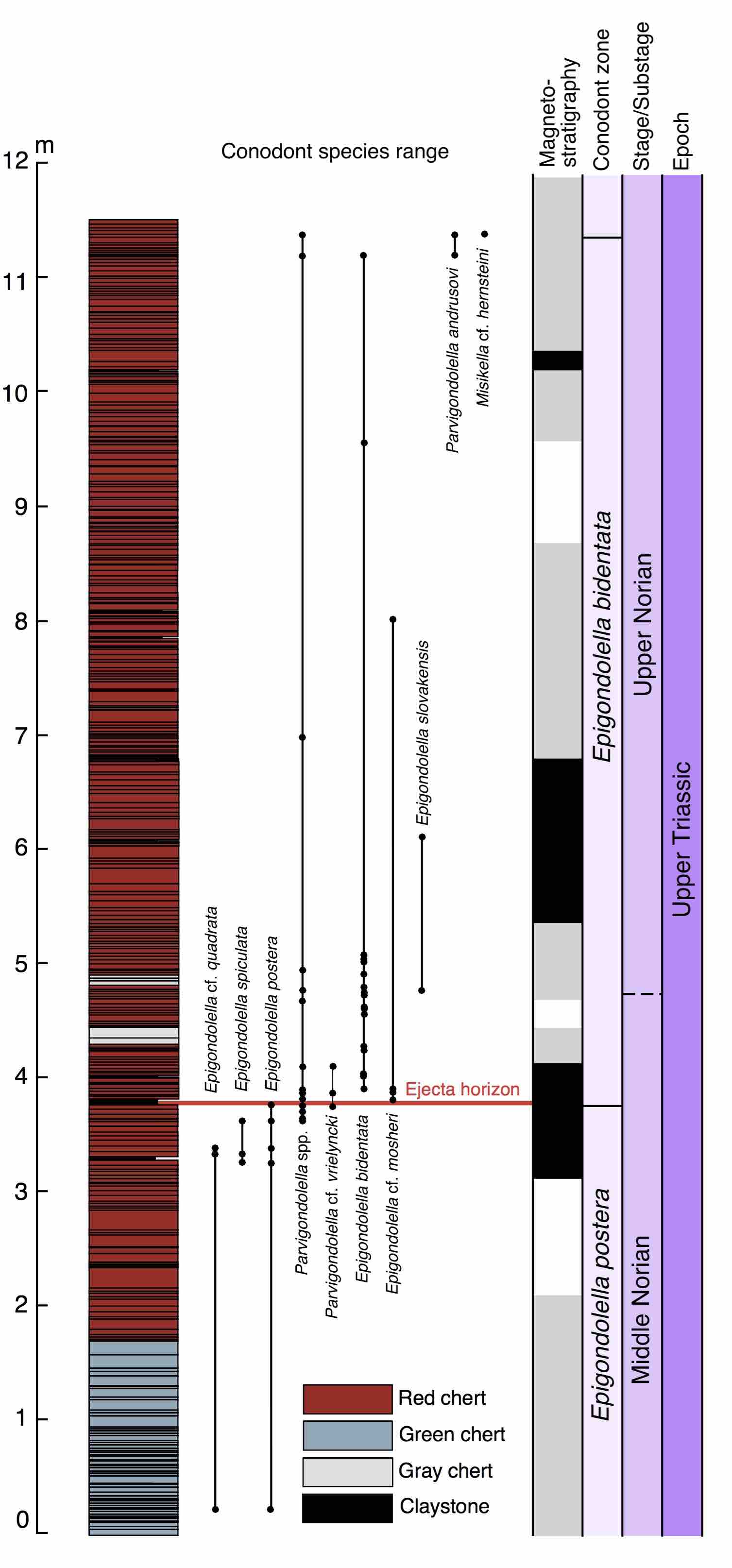
**

**Supplementary Figure S11.** Stratigraphic ranges of Late Triassic conodont species in the Sakahogi section. Modified after ref. 11.

**
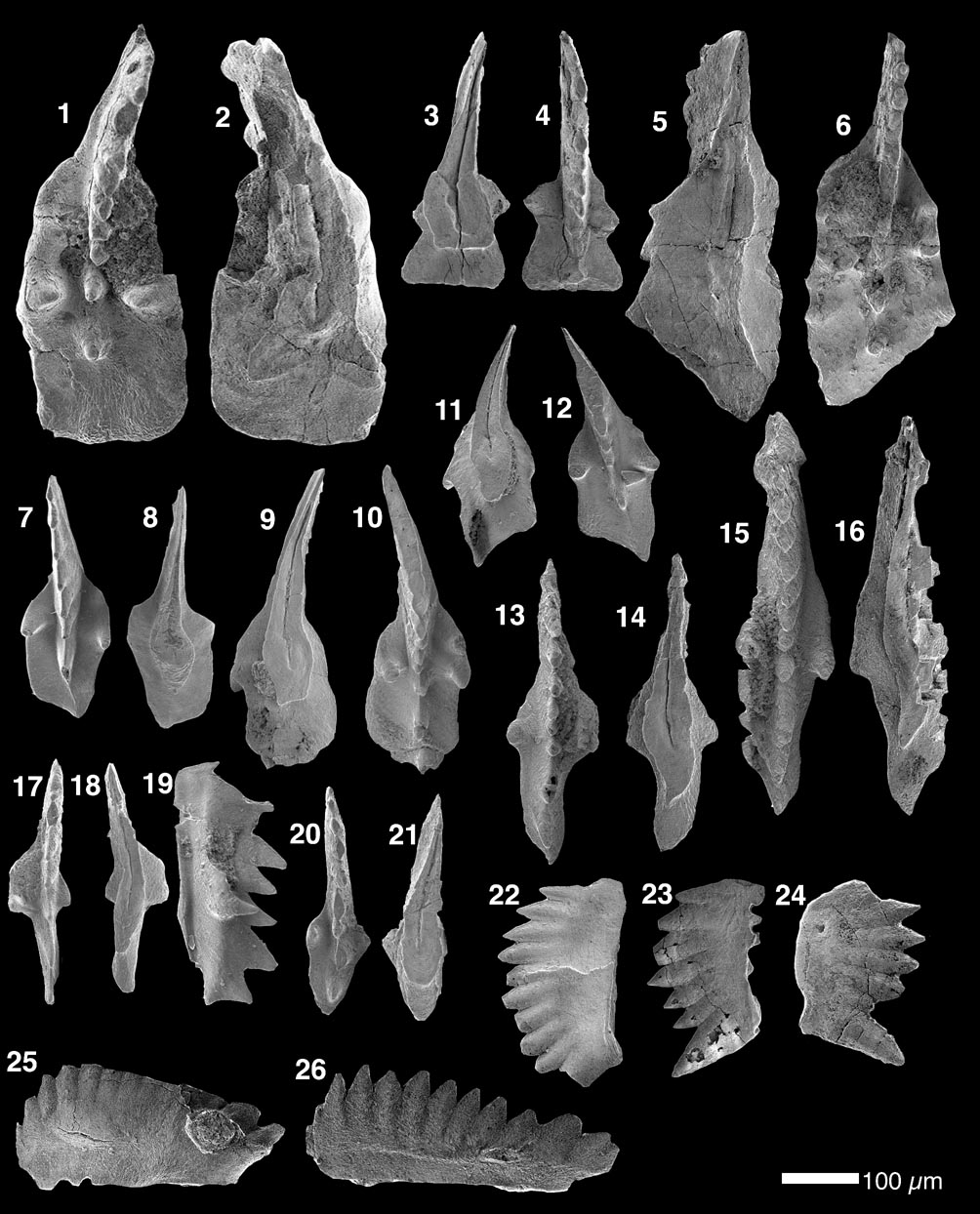
**

**Supplementary Figure S12.** Middle–upper Norian conodonts from the study section. **1–4,** *Epigondolella* sp. cf. *E. quadrata* Orchard. NHR31 (1, 2); NHR30 (3, 4). **5, 6,** *Epigondolella* sp. cf. *E. spiculata* Orchard. NHR37. **7–12,** *Epigondolella postera* Kozur and Mostler. NHR31 (7, 8); NHR40 (9, 10); NHR37 (11,12). **13–16,** *Epigondolella* sp. cf. *E. mosheri* (Kozur and Mostler) Morphotype A Orchard. NHR44 (13, 14, 15, 16). **17–19,** *Epigondolella bidentata* Mosher. NHR45 (17–19). **20, 21,** *Epigondolella* sp. This specimen was previously regarded as *E. bidentata*^7^, but the keel termination of *E. bidentata* is pointed. NHR40 (20, 21). **22–24,** *Parvigondolella* spp. NHR40 (22); NH51 (23); NHR37 (24). **25, 26,** *Parvigondolella* sp. cf. *P. vrielyncki* Kozur and Mock. NHR40 (25); NHR44 (26).

**2. Supplementary Tables**

**Supplementary Table S1.** Measured heights, δ^13^C_org_, total organic carbon (TOC), total nitrogen (TN) values, and C/N ratios of samples from the Sakahogi section.

**Supplementary Table S2.** Major element data for samples from the Sakahogi section. Major element compositions of the Sakahogi chert and claystone samples normalized to 100 wt%.

**Supplementary Table S3.** Estimates of mass accumulation rates (MARs) of biogenic silica is samples from the Sakahogi section.

**Supplementary Table S4.** Explanation of the stratigraphic ranges of radiolarian species, from left to right as displayed in Fig. 3.

1. *Trialatus robustus* (Nakaseko and Nishimura, 1979)
2. *Capnodoce anapetes* De Wever, 1979
3. *Poulpus piabyx* De Wever, 1979
4. *Xiphosphaera fistulata* Carter, 1991
5. *Corum regium* Blome, 1984
6. *Japonocampe nova* (Yao, 1982)
7. *Capnodoce crystallina* Pessagno, 1979
8. *Capnodoce extenta* Blome, 1983
9. *Sepsagon longispinosus* (Kozur and Mostler, 1979)
10. *Syringocapsa batodes* De Wever, 1979
11. *Capnodoce ruesti* Kozur and Mock, 1981
12. *Capnodoce sarisa* De Wever, 1979
13. *Sarla hadrecaena* (De Wever, 1979)
14. *Capnuchosphaera deweveri* Kozur and Mostler, 1979
15. Spumellaria gen. et sp. indet. A
16. *Sarla prietoensis* Pessagno, 1979
17. *Pentactinocarpus sevaticus* Kozur and Mostler, 1981
18. *Palaeosaturnalis largus* (Blome, 1984)
19. *Palaeosaturnalis harrisonensis* (Blome, 1984)
20. *Palaeosaturnalis* sp. aff. *P. harrisonensis* (Blome, 1984)
21. *Palaeosaturnalis* sp. aff. *P. dotti* (Blome, 1984)
22. *Pseudoheliodiscus finchi* Pessagno, 1979
23. *Palaeosaturnalis dotti* (Blome, 1984)
24. *Plafkerium* sp. A
25. *Sarla* sp. B
26. *Pseudoheliodiscus heisseli* (Kozur and Mostler, 1972)
27. *Plafkerium* (?) sp. B
28. *Lysemelas olbia* Sugiyama, 1997
29. *Discofulmen* sp.

**Supplementary Table S5.** Explanation of the stratigraphic ranges of radiolarian species, from left to right as displayed in Supplementary Fig. S4.

1. *Cenosphaera clathrata* Parona, 1890

2. *Paronaella trammeri* (Kozur and Mostler, 1978)

3. *Triassocampe campanilis* (Kozur and Mostler, 1994)

4. *Praemesosaturnalis* (?) *ormites* Sugiyama, 1997

5. *Pseudostylosphaera longispinosa* Kozur and Mostler, 1981

6. *Poulpus* sp. A Sugiyama, 1997

7. *Sarla delicata* Blome, 1983

8. *Sarla* (?) *externa* Blome, 1983

9. *Capnuchosphaera crassa* Yeh, 1990

10. *Capnuchosphaera contracta* Yeh, 1990

11. *Trialatus pristinus* Sugiyama, 1997

12. *Hozmadia* (?) sp. A Sugiyama, 1997

13. *Multimonilis* sp. B Sugiyama, 1997

14. *Trialatus megacornutus* Yeh, 1990

15. *Laxtorum* (?) *carnicum* Sugiyama, 1997

16. *Capnuchosphaera palawanensis* Yeh, 1990

17. *Poulpus phasmatodes* De Wever, 1979

18. *Neopylentonema procera* Sugiyama, 1997

19. *Triassocampe postdeweveri* Kozur and Mostler, 1994

20. *Kahlerosphaera* sp. B Sugiyama, 1997

21. *Veles vulgaris* Sugiyama, 1997

22. *Multimonilis japonicus* Sugiyama, 1997

23. *Capnuchosphaera lea* De Wever, 1979

24. *Enoplocampe yehae* Sugiyama, 1997

25. *Capnuchosphaera sagaris* Sugiyama, 1997

26. *Poulpus* (?) *coronensis* (Yeh, 1990)

27. *Poulpus carcharus* Sugiyama, 1997

28. *Trialatus praerobustus* Sugiyama, 1997

29. *Kahlerosphaera norica* Kozur and Mock, 1981

30. *Trialatus longicornutus* Yeh, 1990

31. *Kahlerosphaera* sp. C Sugiyama, 1997

32. *Xipha nodosa* Sugiyama, 1997

33. *Xiphotheca karpenissionensis* De Wever, 1979

34. *Pachus* (?) *indistinctus* Blome, 1984

35. *Annulopoulpus* sp. A Sugiyama, 1997

36. *Corum* (?) *delgado* Sugiyama, 1997

37. *Kahlerosphaera* sp. A Sugiyama, 1997

38. *Spinopoulpus noricus* Kozur and Mock, 1981

39. *Tritortis kretaensis* (Kozur and Krahl, 1984)

40. *Palaeosaturnalis* (?) *incomptus* Sugiyama, 1997

Table S5 (continued).

41. *Palaeosaturnalis* (?) sp. aff. *P.* (?) *incomptus* Sugiyama, 1997

42. *Capnuchosphaera triassica* De Wever, 1979

43. *Capnuchosphaera oma* Sugiyama, 1997

44. *Capnuchosphaera theloides* De Wever, 1979

45. *Haeckelicyrtium* sp. A Sugiyama, 1997

46. *Capnuchosphaera tricornis* De Wever, 1979

47. *Triassocampe baldii* (Kozur, 1994)

48. *Sarla transita* (Kozur and Mock, 1981)

49. *Canesium lentum* Blome, 1984

50. *Xipha pessagnoi* (Nakaseko and Nishimura, 1979)

51. *Triassocampe proprium* Blome, 1984

52. *Trialatus robustus* (Nakaseko and Nishimura, 1979)

53. *Capnodoce ruesti* Kozur and Mock, 1981

54. *Capnodoce extenta* Blome, 1983

55. *Capnodoce anapetes* De Wever, 1979

56. *Stichocapsa* sp. cf. *S. nana* Sheng, 1976

57. *Dreyericyrtium virgispineum* Sugiyama, 1997

58. *Capnodoce sarisa* De Wever, 1979

59. *Pachus firmus* Blome, 1984

60. *Xiphosphaera fistulata* Carter, 1991

61. *Plafkerium* sp. cf. *P. abbotti* Pessagno, 1979

62. *Poulpus piabyx* De Wever, 1979

63. *Corum regium* Blome, 1984

64. *Capnuchosphaera silviesensis* Blome, 1983

65. *Capnuchosphaera colemani* Blome, 1983

66. *Capnuchosphaera deweveri* Kozur and Mostler, 1979

67. *Sarla hadrecaena* (De Wever, 1979)

68. *Capnodoce crystallina* Pessagno, 1979

69. *Japonocampe nova* (Yao, 1982)

70. *Palaeosaturnalis* sp. aff. *P. dotti* (Blome, 1984)

71. *Syringocapsa batodes* De Wever, 1979

72. *Pseudoheliodiscus finchi* Pessagno, 1979

73. *Palaeosaturnalis largus* (Blome, 1984)

74. *Pentactinocarpus sevaticus* Kozur and Mostler, 1981

75. *Neopaurinella sevatica* Kozur and Mostler, 1981

76. *Pseudoheliodiscus huangi* Yeh, 1990

77. *Archaeosemantis gigas* Sugiyama, 1997

78. *Sarla prietoensis* Pessagno, 1979

79. *Haeckelicyrtium* sp. B Sugiyama, 1997

80. *Dumitricaella parva* Sugiyama, 1997

81. *Mesosaturnalis* sp. aff. *M. artus* (Donofrio and Mostler, 1978)

Table S5 (continued).

82. *Pseudoheliodiscus heisseli* (Kozur and Mostler, 1972)

83. *Mesosaturnalis artus* (Donofrio and Mostler, 1978)

84. *Mesosaturnalis octospinus* Sugiyama, 1997

85. *Archaeosemantis globus* Sugiyama, 1997

86. *Bipedis durus* Sugiyama, 1997

87. *Cantalum* (?) sp. A Sugiyama, 1997

88. *Bipedis triassicus* Yeh and Cheng, 1996

89. *Jacus* sp. aff. *J. isa* De Wever, 1982

90. *Blomella megasphaera* Sugiyama, 1997

91. *Praemesosaturnalis rugosus* Yeh, 1990

92. *Bipedis* sp. aff. *B. acrostylus* Bragin, 1991

93. *Praemesosaturnalis decilobum* (Carter, 1993)

94. *Ayrtonius elizabethae* Sugiyama, 1997

95. *Paronaella* (?) sp. cf. *P.* (?) *beatrica* Carter, 1993

96. *Veghia* sp. A Sugiyama, 1997

97. *Archaeosemantis pterostephanus* Dumitrica, 1978

98. *Braginella rudis* (Bragin, 1968)

99. *Capnuchosphaera neosagaris* Sugiyama, 1997

100. *Annulopoulpus* sp. B Sugiyama, 1997

101. *Paronaella* (?) sp. A Sugiyama, 1997

102. *Archaeosemantis cristianensis* Dumitrica, 1982

103. *Praemesosaturnalis multidentatus* (Kozur and Mostler, 1972)

104. *Sarla vetusta* Pessagno, 1979

105. *Tetraporobrachia composita* Carter, 1993

106. *Archaeosemantis lithocircites* Sugiyama, 1997

107. *Praemesosaturnalis* sp. A Sugiyama, 1997

108. *Lysemelas olbia* Sugiyama, 1997

109. *Praemesosaturnalis pseudokahleri* Sugiyama, 1997

110. *Betraccium deweveri* Pessagno and Blome, 1980

111. *Betraccium yakounense* Pessagno and Blome, 1980

112. *Haeckelicyrtium teren* Sugiyama, 1997

113. *Cantalum* (?) sp. B Sugiyama, 1997

114. *Praemesosaturnalis gracilis* (Kozur and Mostler, 1972)

115. *Praemesosaturnalis bifidus* (Kozur and Mostler, 1972)

116. *Hagiastrum* (?) *pacificum* Sugiyama, 1997

117. *Betraccium* sp. cf. *B. maclearni* Pessagno and Blome, 1980

118. *Praemesosaturnalis zhangi* Yang and Mizutani, 1991

119. *Livarella densiporata* Kozur and Mostler, 1981

120. *Globolaxtorum hullae* Yeh and Cheng, 1996

121. *Pantanellium* sp. cf. *P. newkluense* Carter, 1993

122. *Dreyericyrtium carterae* Bragin and Krylov, 1999

123. *Eptingium* (?) *amoenum* Carter, 1993

Table S5 (continued).

124. *Squinabolella* (?) *trispinosa* Carter, 1993

125. *Praemesosaturnalis* sp. B Sugiyama, 1997

126. *Praemesosaturnalis shengi* Yang and Mizutani, 1991

127. *Paronaella pacofiensis* Carter, 1993

128. *Haeckelicyrtium takemurai* Yeh and Cheng, 1996

129. *Betraccium inornatum* Blome, 1984

130. *Bipedis acrostylus* Bragin, 1991

131. *Dreyericyrtium ithacanthum* Sugiyama, 1997

132. *Risella tledoensis* Carter, 1993

133. *Parvibrachiale yaoi* Sugiyama, 1997

134. *Livarella valida* Yoshida, 1986

135. *Haeckelicyrtium breviora* Sugiyama, 1997

136. *Pseudohagiastrum giganteum* Carter and Hori, 2005

137. *Globolaxtorum tozeri* Carter, 1993

138. Spumellaria gen. et sp. Indet. A Carter and Hori, 2005

139. *Praehexasaturnalis tenuispinosus* (Donofrio and Mostler, 1978)

140. *Amuria impensa* Whalen and Carter, 1998

141. *Archaeocenosphaera laseekensis* Pessagno et al., 1989

142. *Pseudacanthocircus* sp. C Sugiyama, 1997

143. *Paratriassoastrum* sp. cf. *P. crassum* Carter, 1993

144. Spongotrochus? sp. (Carter, 1994)

145. *Pentaspongodiscus* (?) *dihexacanthus* Carter, 1993

146. *Palaeosaturnalis harrisonensis* (Blome, 1984)

147. *Citriduma asteroides* Carter, 1993

148. *Palaeosaturnalis* sp. aff. *P. harrisonensis* (Blome, 1984)

149. *Praemesosaturnalis heilongjiangensis* Yang and Mizutani, 1991

150. *Canoptum rhaeticum* Kozur and Mostler, 1981

151. *Palaeosaturnalis dottoi* (Blome, 1984)

152. *Pseudacanthocircus* sp. B Sugiyama, 1997

153. *Pseudacanthocircus pseudosimplex* Kozur and Mostler, 1990

154. *Pantanellium tanuense* Pessagno and Blome, 1980

155. *Pantanellium* aff. *browni* Pessagno and Blome, 1980

156. *Bipedis* cf. *hannai* Carter et al., 1998
